# Community paramedic hospital reduction and mitigation program: study protocol for a randomized pragmatic clinical trial

**DOI:** 10.1186/s13063-022-07034-w

**Published:** 2023-02-20

**Authors:** Jennifer L. Ridgeway, Erin O. Wissler Gerdes, Andrew Dodge, Chad P. Liedl, Michael B. Juntunen, Wendy J. S. Sundt, Amy Glasgow, Michelle A. Lampman, Angela L. Fink, Sara B. Severson, Grace Lin, Richard R. Sampson, Robert P. Peterson, Brian M. Murley, Aaron B. Klassen, Anuradha Luke, Paul A. Friedman, Tamara E. Buechler, James S. Newman, Rozalina G. McCoy

**Affiliations:** 1 Division of Health Care Delivery Research, Robert D. and Patricia E. Kern Center for the Science of Health Care Delivery, Mayo Clinic, Rochester, MN USA; 2 Robert D. and Patricia E. Kern Center for the Science of Health Care Delivery, Mayo Clinic, Rochester, MN USA; 3 Mayo Clinic Ambulance, Rochester, MN USA; 4 Research Services – Clinical Trials Office, Mayo Clinic, Rochester, MN USA; 5 Department of Cardiovascular Medicine, Mayo Clinic, Rochester, MN USA; 6 Department of Family Medicine, Mayo Clinic Health System - Northland, Barron, WI USA; 7 Division of Hospital Internal Medicine, Mayo Clinic Health System - Northland, Barron, WI USA; 8 Department of Emergency Medicine, Mayo Clinic Ambulance, Rochester, MN USA; 9 Division of Hospital Internal Medicine, Mayo Clinic, Rochester, MN USA; 10 Department of Medicine, Division of Community Internal Medicine, Geriatrics, and Palliative Care, Rochester, MN USA

**Keywords:** Emergency medical services, Community paramedicine, Mobile integrated health, Readmission, Health care delivery, Home health, Rural, Community health services, Patient-centered care, Pragmatic clinical trial, Implementation science

## Abstract

**Background:**

New patient-centered models of care are needed to individualize care and reduce high-cost care, including emergency department (ED) visits and hospitalizations for low- and intermediate-acuity conditions that could be managed outside the hospital setting. Community paramedics (CPs) have advanced training in low- and high-acuity care and are equipped to manage a wide range of health conditions, deliver patient education, and address social determinants of health in the home setting. The objective of this trial is to evaluate the effectiveness and implementation of the Care Anywhere with Community Paramedics (CACP) program with respect to shortening and preventing acute care utilization.

**Methods:**

This is a pragmatic, hybrid type 1, two-group, parallel-arm, 1:1 randomized clinical trial of CACP versus usual care that includes formative evaluation methods and assessment of implementation outcomes. It is being conducted in two sites in the US Midwest, which include small metropolitan areas and rural areas. Eligible patients are ≥ 18 years old; referred from an outpatient, ED, or hospital setting; clinically appropriate for ambulatory care with CP support; and residing within CP service areas of the referral sites. Aim 1 uses formative data collection with key clinical stakeholders and rapid qualitative analysis to identify potential facilitators/barriers to implementation and refine workflows in the 3-month period before trial enrollment commences (i.e., pre-implementation). Aim 2 uses mixed methods to evaluate CACP effectiveness, compared to usual care, by the number of days spent alive outside of the ED or hospital during the first 30 days following randomization (primary outcome), as well as self-reported quality of life and treatment burden, emergency medical services use, ED visits, hospitalizations, skilled nursing facility utilization, and adverse events (secondary outcomes). Implementation outcomes will be measured using the RE-AIM framework and include an assessment of perceived sustainability and metrics on equity in implementation. Aim 3 uses qualitative methods to understand patient, CP, and health care team perceptions of the intervention and recommendations for further refinement. In an effort to conduct a rigorous evaluation but also speed translation to practice, the planned duration of the trial is 15 months from the study launch to the end of enrollment.

**Discussion:**

This study will provide robust and timely evidence for the effectiveness of the CACP program, which may pave the way for large-scale implementation. Implementation outcomes will inform any needed refinements and best practices for scale-up and sustainability.

**Trial registration:**

ClinicalTrials.gov NCT05232799. Registered on 10 February 2022.

## Administrative information

Note: The numbers in curly brackets in this protocol refer to the SPIRIT checklist item numbers. The order of the items has been modified to group similar items (see http://www.equator-network.org/reporting-guidelines/spirit-2013-statement-defining-standard-protocol-items-for-clinical-trials/).

**Table:** 

Title {1}	Community paramedic hospital reduction and mitigation program: study protocol for a randomized pragmatic clinical trial
Trial registration {2a and 2b}.	Registered in ClinicalTrials.gov NCT05232799. Item 2b is met if the register used for registration collects all items from the World Health Organization Trial Registration Data Set.
Protocol version {3}	June 10, 2022, Version 4
Funding {4}	This study was funded by Mayo Clinic Clinical Trials Award Funding.
Author details {5a}	•Jennifer L. Ridgeway; Division of Health Care Delivery Research; Robert D. and Patricia E. Kern Center for the Science of Health Care Delivery, Mayo Clinic, Rochester, MN •Erin O. Wissler Gerdes; Robert D. and Patricia E. Kern Center for the Science of Health Care Delivery, Mayo Clinic, Rochester, MN •Andrew Dodge; Robert D. and Patricia E. Kern Center for the Science of Health Care Delivery, Mayo Clinic, Rochester, MN •Chad P. Liedl; Mayo Clinic Ambulance, Rochester, MN •Michael B. Juntunen; Mayo Clinic Ambulance, Rochester, MN •Wendy JS Sundt; Research Services – Clinical Trials Office, Mayo Clinic, Rochester, MN •Amy Glasgow; Robert D. and Patricia E. Kern Center for the Science of Health Care Delivery, Mayo Clinic, Rochester, MN •Michelle A. Lampman; Robert D. and Patricia E. Kern Center for the Science of Health Care Delivery, Mayo Clinic, Rochester, MN •Angela L. Fink; Department of Cardiovascular Medicine, Mayo Clinic, Rochester, MN •Sara B. Severson; Department of Cardiovascular Medicine, Mayo Clinic, Rochester, MN •Grace Lin; Department of Cardiovascular Medicine, Mayo Clinic, Rochester, MN •Richard R. Sampson; Department of Family Medicine, Mayo Clinic Health System—Northland, Barron, WI •Robert P. Peterson; Division of Hospital Internal Medicine, Mayo Clinic Health System—Northland, Barron, WI •Brian M. Murley; Mayo Clinic Ambulance, Rochester, MN •Aaron B Klassen; Department of Emergency Medicine; Mayo Clinic Ambulance, Rochester, MN •Anuradha Luke; Department of Emergency Medicine; Mayo Clinic Ambulance, Rochester, MN •Paul A. Friedman; Cardiovascular Medicine, Mayo Clinic, Rochester, MN •Tamara E. Buechler; Division of Hospital Internal Medicine, Mayo Clinic, Rochester, MN •James S. Newman; Division of Hospital Internal Medicine, Mayo Clinic, Rochester, MN •Rozalina G. McCoy; Division of Health Care Delivery; Robert D. and Patricia E. Kern Center for the Science of Health Care Delivery; Mayo Clinic Ambulance; Division of Community Internal Medicine, Geriatrics, and Palliative Care, Department of Medicine, Rochester, MN
Name and contact information for the trial sponsor {5b}	This study was supported by Grant Number UL1 TR002377 from the National Center for Advancing Translational Sciences (NCATS) and the Mayo Clinic Robert D. and Patricia E. Kern Center for the Science of Health Care Delivery.
Role of sponsor {5c}	The funders and sponsors had no role in the design of the study and will have no role in the data collection, analysis, or interpretation and reporting, including the final decision to submit the report for publication.

## Introduction

### Background and rationale {6a}

Individualized patient-centered care should deliver the right level of care, by health care providers working at the top of their licensure and at a location most suited to the patient’s needs, goals, and preferences. This approach to patient care spans the hospital and home environments, leverages interdisciplinary teams, and adapts care delivery models to the needs and circumstances of each patient. One high-priority area for patients, hospitals, payers, and the health care system is preventing and shortening hospitalizations. Hospitalizations are a major contributor to high costs of care, are highly resource-intensive and resource-limited, are not equitably available (particularly in rural areas), and contribute to patients’ illness burden.

Historically, the continuum of care has been fragmented into discrete elements delivered in the home (typically limited to conditions managed with oral therapies and without close monitoring), emergency department (ED) or hospital observation unit (conditions requiring short duration of more invasive therapies and/or monitoring), and hospital inpatient units (conditions requiring a longer duration of invasive therapies and/or monitoring). Often, patients are referred to the ED and/or hospitalized for intermediate acuity conditions or for conditions that require limited interventions because the necessary level of care (e.g., intravenous [IV] therapies, wound care, education of patients and caregivers) is unavailable in the home.

Innovative care delivery models that leverage multidisciplinary teams may help health care systems better meet the full continuum of patient care needs effectively, efficiently, and equitably. However, the “missing link” in many efforts to treat patients at home and prevent hospitalizations has been the absence of a team that could effectively, safely, and efficiently deliver a wide range of services in the patient’s home as would be necessary to prevent or shorten an ED visit or hospitalization. Community paramedics (CPs) are paramedics with advanced training in primary and preventive care, chronic disease management, and social determinants of health. Community paramedicine is an innovative care delivery model that provides high-quality, cost-effective primary and disease-directed care [1, 2] in the community setting using specialty-trained paramedics under the supervision of a physician medical director. To date, most CP programs have focused on supporting transitions of care among patients with a history of frequent hospital, ED, and/or EMS utilization or with multi-morbidity [3–10], with the goals of reducing ED visits and hospitalizations [3–5, 7, 11–14] and connecting patients to social, community, and medical services [3, 15].

Building on this emerging model of care, the goal of this study is to evaluate the effectiveness and implementation of an innovative, generalizable, and adaptive program that leverages the unique skillset of CPs to reduce health care utilization among patients requiring an *intermediate level of care*. These include patients who are hospitalized or in the ED but could be discharged to the ambulatory setting with CP support (goal of reducing the length of stay [LOS]) or who are in the ambulatory setting but likely to be hospitalized or referred to ED but could avoid hospitalization with CP support (goal of preventing ED visits and/or hospitalizations).

### Objectives {7}

The primary objective of this trial is to evaluate the effectiveness and safety of the Care Anywhere with Community Paramedics (CACP) program with respect to increasing days alive spent outside the ED or hospital between the day after randomization and 30 days thereafter among patients (1) being treated in the ED or hospital but clinically stable for ambulatory care with CP support and (2) being treated in the pre-hospital setting (e.g., outpatient clinic or home) but requiring a higher level of care that would usually prompt a referral to the ED/hospital but could be managed by the CACP program. Secondarily, we will examine the CACP program with respect to reach, adoption, acceptability, implementation, and sustainability from the patient, CP, referring clinician, and health system administrator perspectives; patient satisfaction with the program; patient experience with treatment and self-management, i.e., treatment burden; patient safety outcomes at 30 days (falls, medication errors, ED visits, hospitalizations, death); and long-term acute care utilization (i.e., ED visits, hospitalizations) outcomes at 6 and 12 months. This hybrid approach to exploring implementability preparatory to and during an effectiveness trial is aimed at identifying implementation issues so strategies to address them can be incorporated into trial adaptations or future implementation [16, 17].

There are three study aims:

- Aim 1: Identify potential facilitators and barriers to implementation and refine workflows in pre-implementation phase
- Aim 2: Evaluate CACP effectiveness and safety, compared to usual care, as well as implementation outcomes
- Aim 3: Assess patient, CP, clinician, and administrator acceptability, satisfaction, and perceived sustainability

### Trial design {8}

This study is a pragmatic, hybrid type 1, two-group, parallel-arm, 1:1 randomized clinical trial. It includes formative evaluation and implementation outcomes, but as a type 1 hybrid trial, the primary focus is on effectiveness [16, 17]. Aim 1 formative evaluation will take place during the 3-month period before trial enrollment commences (i.e., pre-implementation) and will use qualitative data collection with a range of clinical and administrative stakeholders to identify potential facilitators and barriers to implementation and to refine workflows, ensuring feasible implementation and study conduct. Aim 2 will use data from the electronic health record (EHR) and surveys to evaluate CACP effectiveness and safety, compared to usual care, by the number of days spent alive outside of the ED or hospital during the first 30 days following randomization (primary outcome), as well as to evaluate EHR-derived and patient-reported outcomes, including quality of life (QoL), and experience with treatment and self-management (i.e., treatment burden). Implementation outcomes will be evaluated using the RE-AIM framework and will include process measures from administrative data (e.g., dropout rates and reasons by rurality, race/ethnicity, age, and gender) [18]. Aim 3 will use qualitative interviews and surveys with patients, CPs, and members of the health care team (clinicians, administrators) to assess acceptability/satisfaction and perceived sustainability, which are critical for moving from the pragmatic trial to enduring practice change and further scalability.

## Methods: participants, interventions, and outcomes

### Study setting {9}

**Fig. 1:**
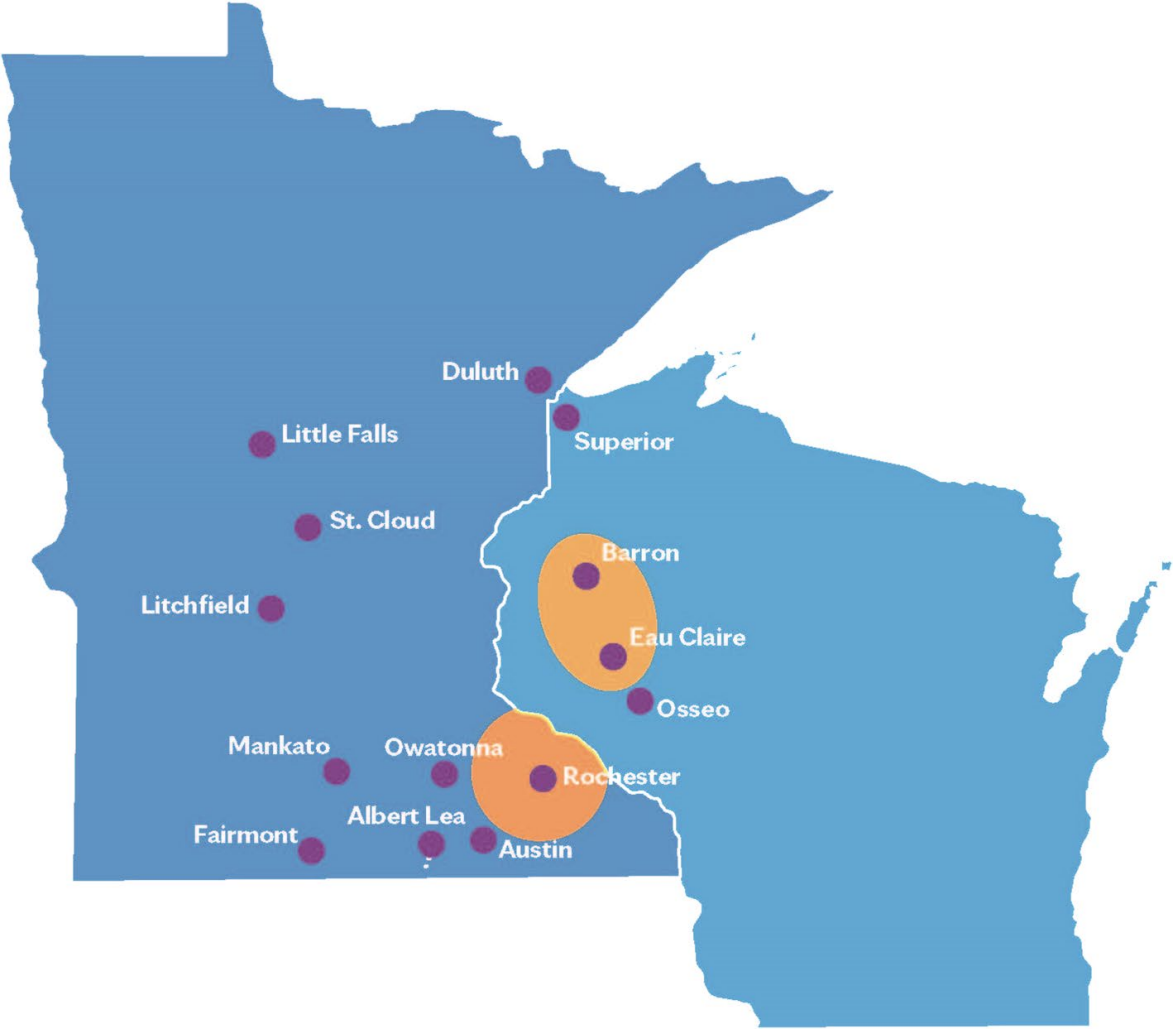
CACP program service areas. Approximate service areas for the CACP program are illustrated in orange. The service areas are based on the affiliated clinical locations. The first is in Minnesota and includes an approximately 40-mile area surrounding Rochester, MN, USA. The second is in Wisconsin and includes the area between and around Eau Claire and Barron, WI, USA

This study will take place with patients who are referred from the pre-hospital (e.g., outpatient clinic or home), ED, or hospital settings in Rochester, MN, USA, and the Northwest region of Wisconsin, USA, and whose home environment for care is within the service areas of the CACP program, as shown in Fig. 1.

### Eligibility criteria {10}

The following are the inclusion criteria:

- Aim 1: Clinicians (physicians, nurse practitioners, physician assistants), nurses, CPs, administrators, case managers, social workers, and other stakeholders likely to be engaged in or impacted by the CACP program
- Aim 2: Adults (age ≥ 18 years) who are (1) currently admitted to the ED or hospital or (2) in a pre-hospital (i.e., outpatient) setting but being considered for referral to the ED or hospital and who do not require hospital-level monitoring or care other than services that could be delivered by CPs in the ambulatory setting if such services were available to them
- Aim 3: Patients enrolled in the CACP program, CPs affiliated with the CACP program, health care team members (clinicians, case managers, social workers) who referred participants enrolled in the CACP intervention arm, and administrators involved with the CACP program or affiliated programs and services

The following are the exclusion criteria:

- Aim 1: No exclusion criteria.
- Aim 2: Patients will be excluded if (1) the referring clinician believes they require ED or hospital care; (2) they or their legal guardian/representative are unable or unwilling to give written informed consent; (3) they demonstrate clinical, behavioral, or cognitive instability, as determined by the referring clinician or the CACP service; (4) their living conditions are deemed unsafe for CPs to enter (e.g., the patient refuses to secure weapons or animals prior to CP arrival); (5) they were enrolled in the trial during an earlier hospitalization or ED visit; or (6) they are requesting services in a location outside the CACP service area or in a skilled nursing facility (SNF). The referring service completes a checklist prior to referring patients to the CACP program (available as a system smart phrase within the EHR) that explains these criteria, including patient confirmation of factors related to the safety of CPs conducting home visits. Patients can only be enrolled in the trial if the CACP program has the census capacity to accept new patients.
- Aim 3: There are no exclusion criteria for clinicians or other clinical or administrative stakeholders. Patient participants will be excluded from recruitment to interviews if they (1) have a communication barrier due to medical illness or cognitive impairment or (2) are seriously ill or hospitalized at the time of interview recruitment.

### Who will take informed consent? {26a}

For aim 1, a member of the study team will contact individuals who have been identified as being in a role that will potentially interact with the CACP program or be impacted by the program. Individuals who agree to participate in a qualitative interview will complete oral consent procedures prior to the start of the interview. For aim 2, the referring service will introduce the CACP program to patients they deem to be in need of CP services and gauge the patient’s interest in the CACP program and the research trial evaluating the program. If the patient is interested, the referring service will contact the CACP program using a clinical referral pathway. The CACP program team will screen patients for clinical appropriateness and verify the program capacity to accept new patients. If the patient is appropriate for CP care and the program has the capacity to deliver the requested services, a member of the research team will be notified to approach and consent the patient. Consent will be obtained in person, by telephone, or by video conference, depending on the location of the referral. The study team member will detail the research study as well as the intervention and verify that the patient meets the eligibility criteria. Once eligibility is confirmed, the participant will provide consent via the signature of an electronic consent form (for remote consent) or sign the paper copy of the consent document (for in-person consent and remote consent without electronic access). In situations where remote consent is necessary but the patient does not have access to an electronic device to provide electronic consent, a PDF of the prepared consent form will be emailed via secure institutional email to a member of the patient’s clinical team; they will hand two copies of the consent document to the patient and will collect the signed form from them after consent is obtained via telephone by the research team (the referring service will not seek consent themselves). For aim 3, participants will complete oral consent procedures prior to their interview. For surveys, a cover letter will include an explanation of the potential risks and benefits of participation, and it will specify that participation is voluntary and will not influence employment or education at Mayo Clinic. Consent to participate will be indicated by survey completion.

### Additional consent provisions for collection and use of participant data and biological specimens {26b}

The consent process states that de-identified information gathered as part of this study may be used for future research or shared with other researchers without additional informed consent. Handling of biological specimens and plans for future ancillary studies using biological samples are not applicable to this trial. Any future studies using participant data from this trial will be reviewed by the affiliated Institutional Review Board.

### Interventions

#### Explanation for the choice of comparators {6b}

This trial compares CP care to usual care. This comparison is suitable for a pragmatic trial that aims to generate evidence in real-world settings. The usual care condition is reflective of the most common approach to care in similar types of settings, which will allow for the generalizability of findings.

#### Intervention description {11a}

The CACP program is an extension of an established care delivery model offered by the Mayo Clinic Ambulance Service. In the CACP program, the referring clinician identifies services or interventions clinically required for the patient. This may include patient education, clinical reassessment, laboratory specimen collection, medication administration, wound care, and other services within the CP scope of service. Visits are limited to up to two per day and are generally no more than 2 h in duration. The referring service or the patient’s primary care provider (PCP) or another ambulatory care provider, as appropriate, oversees clinical care with the support of the CP medical director. CPs will communicate with the referring service and/or their medical director as appropriate (usually after every visit but individualized based on the patient’s need and instructions provided by the referring service) via EHR inbox messages, the EHR chat function, or telephone. Patients will graduate from the program once the goals outlined in the referral are completed. Upon graduation, the CP team will communicate directly with a clinician assuming the patient’s care (e.g., PCP).

The CACP program will be available to patients at no cost, and services provided by CPs under this trial will not be submitted to participating patients’ insurance. If CPs facilitate or assist with a service performed by other members of the health care system, the services performed by others will be processed by the patient’s insurance as per usual procedures. For example, if a patient is seen for clinical assessment, venipuncture with laboratory sample collection, and administration of intravenous fluids, the assessment, venipuncture, IV start, and IV fluids themselves will be free to the patient. However, the analysis of the blood sample conducted by a clinical laboratory after it is collected and delivered by CPs will be processed through insurance. In contrast, point-of-care testing performed by CPs will be at no cost to the patient.

Participants randomized to usual care will be treated without consideration of CP care options. We anticipate that most will be referred to the ED and admitted to the hospital or have alternative care pathways (e.g., home health agency, SNF, frequent clinic visits) to meet their clinical needs. The start time for both cohorts will be the day after randomization.

#### Criteria for discontinuing or modifying allocated interventions {11b}

Care in the intervention and usual care arms are available in standard clinical practices. Participants can withdraw from the intervention voluntarily at any time. Any participants in the intervention arm who require ED, hospital care, or skilled nursing facilities after randomization will continue in the intervention arm such that health care utilization can be assessed as trial outcomes.

#### Strategies to improve adherence to interventions {11c}

For participants randomized to CP care, primary adherence is defined as completing the first CP visit. Secondary adherence is defined as completing subsequently scheduled CP visits. Strategies to improve adherence include specifying the time of the first CP visit on the patient’s hospital or ED discharge summaries (if this information is available at the time of discharge), including CP visits on the patient’s clinic appointment schedule and online portal, and conducting reminder phone calls prior to the visit.

#### Relevant concomitant care permitted or prohibited during the trial {11d}

Both study arms will be executed per usual clinical practice, consistent with the pragmatic study design. As such, there are no restrictions on concomitant interventions.

#### Provisions for post-trial care {30}

The need for ancillary and post-trial care is not anticipated as part of this trial. For participants in the intervention arm, the provision of any ancillary care during the trial, including that related to the needs identified by CPs, will be managed and/or compensated using standard patient procedures. After the outcome ascertainment period is completed (day 30), if study participants require CP services, they will be eligible to receive them through the usual clinical care pathway irrespective of study allocation.

### Outcomes {12}

Trial outcomes for the three specific aims are detailed in Tables 1 and 2. Aim 1 is a formative evaluation of the implementation context that seeks to rapidly identify and address anticipated barriers and challenges, as well as identify opportunities that could impact the successful implementation of the pragmatic trial and the CACP program. It involves qualitative interviews with CPs, clinicians and other members of the health care team, and administrators, in the 3-month period directly before trial enrollment commences with anticipated qualitative outcomes related to acceptability and feasibility, as well as detailed recommendations for targeted actions.

**Table 1:** Elements of primary and secondary outcomes

Domain	Specific measurement	Specific metric	Method of aggregation	Time point
Potential barriers to CACP implementation (aim 1)	Qualitative interviews	Perspectives on acceptability, feasibility, and workflow impact	Content analysis by construct by role, e.g., CP, clinician, administrator	Prior to the start of the enrollment period (− *T*_1_)
CACP effectiveness (aim 2 primary)	EHR review	Days alive out of ED or hospital 0 and *T*_3_	The number of days the patient was alive and out of the ED or hospital (not in hospital, excluding planned hospital admissions for scheduled surgeries, procedures, and/or treatments) between 0 and *T*_3_	Comparison between the groups will be made at *T*_3_
CACP effectiveness (aim 2 secondary)	EHR review	Unplanned ED visits, hospitalizations, or death (composite endpoint and individual)	The number of events per participant per 30 days, the percent of participants with events within 30 days, and time to event(s). Will report as a composite outcome and individually	Comparison between the groups at *T*_4_ and *T*_5_
CACP effectiveness (aim 2 exploratory)	EHR review	SNF care	The percent of participants admitted to SNF within 30 days of randomization and duration of SNF stay	Comparison between the groups will be made at *T*_3_
CACP safety (aim 2 secondary)	EHR review	Unplanned hospitalizations, ED visits, falls with injury, medication errors with harm, and death	The percentage of patients with events within 30 days, the total number of events within 30 days, and time to event(s). Will report as a composite outcome and each outcome individually	Comparison between the groups at *T*_3_ and the day after randomization to *T*_3_
HRQoL (aim 2 secondary)	EQ-5D survey	HRQoL scores (0–100) calculated across five domains	The scores will be summarized using mean, SD, minimum, median, and maximum scores by treatment arm at *T*_3_	Comparison between the groups at *T*_3_ and the day after randomization to *T*_3_
Patient experience with treatment and self-management, i.e., treatment burden (aim 2 secondary)	PETS survey	PETS scores calculated for each domain	Domain scores will be summarized using mean, SD, and minimum and maximum scores	Comparison between the groups at *T*_3_ and the day after randomization to *T*_3_
Patient evaluation of CP care (aim 2 secondary)	Surveys items on involvement in care, communication and health information, caring and concern, and care coordination	Patient-reported CP involvement in care	Percentage of patients who respond “strongly agree” or “agree.”	Descriptive assessment of intervention patients in the CACP program at *T*_2_
Program satisfaction (aim 3 secondary)	Surveys items on program satisfaction	Self-reported satisfaction with CP care and the CACP program	Percentage of respondents who are “extremely satisfied” or “very satisfied.”	Comparison between participant types will be made after enrollment ends
Program satisfaction and recommendations for improvement and sustainability (aim 3 secondary)	Qualitative interviews	Program satisfaction and recommendations on improvement and sustainability	Perspectives on how well the program meets user needs and whether/how it should be continued	*T*_2_ for a sample of patients in the CACP program and after enrollment ends for CPs and referring clinicians

* − T*_*1*_ enrollment, *0* allocation, *T*_*1*_ first CP visit, *T*_*2*_ after last CP visit (note: *T*_2_ can be before or after *T*_3_, depending on the patient’s clinical need and how long they will be receiving CP services), *T*_*3*_ 30 days post-allocation, *T*_*4*_ 6 months post-allocation, *T*_*5*_ 12 months post-allocation

**Table 2:** Implementation outcomes

RE-AIM domain	Outcome	Source	Time point
Reach	1.Percentage of patients referred to CACP who were declined prior to randomization due to program capacity, being outside the catchment area, or scope of practice 2.Percentage of patients randomized to CACP who decline the program or are no longer able to enroll due to change in clinical status 3.Percentage of patients consenting to randomization by factors including rurality, race/ethnicity, gender, and age 4.Dropout rates and reasons by factors including rurality, race/ethnicity, gender, and age	CACP enrollment tracker linked to EHR for patient characteristics	After enrollment ends
Effectiveness	Qualitative data to contextualize when and for whom the program was or was not effective	Interviews with patients, CPs, and referring clinicians	*T*_2_ for CACP patients and after enrollment ends for CPs and referring clinicians
Adoption	1.Number of care teams making referrals and number of referrals made overall and by service line, indication, and patient population (focus on disparities by age, gender, rurality, race/ethnicity) 2.Change in adoption over time	CACP enrollment tracker linked to EHR for patient characteristics	After enrollment ends
Implementation	1.Completed delivery, fidelity, and adaptation 2.Acceptability, feasibility, and barriers and facilitators to implementation 3.Availability of resources to implement by setting 4.Capacity to deliver the intervention the same way with all types of patients in all settings	Adaptation tracking database and interviews with CPs, referring clinicians, and administrators	After enrollment ends
Maintenance	1.Should the program be adapted to meet user needs? 2.Perceptions of program sustainability	CSAT survey and interviews with CPs, referring clinicians, and administrators	Overall summary and comparison by role after enrollment ends

Aim 2 will evaluate CACP effectiveness (compared to usual care) and implementation outcomes using mixed methods. The primary effectiveness outcome is the number of days alive out of ED or hospital within 30 days of the day following randomization, ascertained by EHR review; this outcome includes all unplanned hospitalizations, ED visits, and deaths, which will also be assessed separately as safety endpoints. This primary outcome measure was selected for its relevance to the desired goal of the CACP program, i.e., shortening and/or preventing ED and hospital utilization. It captures both the initial impact of CP services availability and the effectiveness of the program in meeting the needs of the patient and preventing the return to the ED/hospital subsequent to initial discharge. Planned hospital stays will be excluded from the primary outcome. The secondary outcomes include the proportions of patients experiencing the components of the primary outcome (i.e., percent of patients with unplanned hospitalizations, ED visits, and death), the rates of each component of the primary outcome, the proportion of patients requiring SNF care, as well as patient-reported health-related quality of life (HRQoL) and treatment burden using the patient-reported EQ-5D [19] and PETS [20] measures. For patients in the CACP program, patient satisfaction will also be evaluated. Implementation outcomes, organized by the RE-AIM implementation evaluation framework (Reach, Effectiveness, Adoption, Implementation, and Maintenance), as shown in Table 2, will be ascertained using administrative and EHR data and interviews. Reach and adoption outcomes include metrics for the eligible population, while other implementation outcomes are specific to the intervention group. Additional secondary analyses will assess for the heterogeneity of treatment effects as a function of baseline ED/hospital utilization, source of referral, reason for referral, services delivered, patient demographics (age, sex, rurality, race/ethnicity, limited English proficiency, area-level deprivation), number of CP visits, and duration of CP care. Sensitivity analyses will be performed excluding hospice deaths and utilizing the per-protocol rather than the intention-to-treat approach.

Aim 3 will assess acceptability/satisfaction and perceived sustainability using surveys [21] and interviews with patients, CPs, clinicians and other members of the health care team, and administrators.

#### Participant timeline {13}

The schedule of enrollment, intervention, and assessment for patient participants is shown in Table 3. Adults (age ≥ 18 years) being treated in the pre-hospital environment (e.g., outpatient clinic or home), ED, or hospital will be identified by their treating clinicians as needing and being clinically appropriate for outpatient management with supportive services provided by the CP team. Clinicians will query patient interest, and a member of the study team will subsequently approach, consent, and enroll patients. Subsequent to consent, they will be randomized 1:1 to CACP versus continued usual care, with stratification by source of referral (pre-hospital versus ED versus hospital) and location (Minnesota versus Wisconsin, corresponding to the two different CP teams delivering the intervention). Participants randomized to CACP will be eligible to receive CP services as ordered by the referring clinician. Participants randomized to usual care will continue to receive care as they would otherwise, including being referred to the ED (for patients in the pre-hospital setting), admitted to the hospital (for patients in the ED), or remain in the hospital (for patients already in the hospital) as appropriate.

**Table 3:** Flow of study procedures for patient participants

	**Study period**						
	**Enrollment**	**Allocation**	**Post-allocation**				
**Time point**	*** − T***_***1***_	**0**	***T***_***1***_	***T***_***2***_	***T***_***3***_	***T***_***4***_	***T***_***5***_
**Enrollment**							
Eligibility screen	X						
Informed consent	X						
Allocation		X					
**Interventions**							
CACP care			X				
**Assessments**^**a**^							
Age, sex, race, ethnicity, comorbidities, medications (EHR)	X						
Social support information (survey)		X					
HRQoL—EQ-5D (survey)		X			X		
Treatment burden—PETS (survey)		X			X		
ED visits, unplanned hospitalizations, death (EHR)					X	X	X
Falls with injury, medication errors with harm					X		
Program experience (survey and interview^b^)				X			

* − T*_*1*_ enrollment, *0* allocation, *T*_*1*_ first CP visit, *T*_*2*_ after last CP visit (note: *T*_2_ can be before or after *T*_3_, depending on the patient’s clinical need and how long they will be receiving CP services), *T*_*3*_ 30 days post-allocation, *T*_*4*_ 6 months post-allocation, *T*_*5*_ 12 months post-allocation

^a^Assessments are interviews, self-report surveys, and EHR (chart abstraction)

^b^Patients in the CACP program will complete a *T*_2_ survey. A sample of patients in the CACP program will be recruited to participate in an interview

Patient participants will be followed for 1 year post-allocation. They will complete surveys at baseline and 30 days; participants randomized to CACP will also complete surveys after their last CP visit. The EHR will be used to ascertain ED visits and unplanned hospitalizations (e.g., excluding planned hospital admissions for scheduled surgeries, procedures, and/or treatments at 30 days, 6 months, and 12 months post-allocation). If patients received CP services as part of usual care between 30 days and 12 months, this will be identified from the EHR and accounted for in the analyses.

#### Sample size {14}

For aim 1, the sample size will be assessed iteratively during data collection and analysis with the aim of ensuring that the implementation plan is feasible and meets the needs of key user groups. We anticipate up to 25 interviews in the pre-implementation phase for aim 1.

The aim 2 sample size was calculated on the basis of the primary hypothesis. Pilot studies of CP care on days out of ED or hospital in the first 30 days yielded an estimated mean of 27 days in the intervention group. A difference of 2 days was considered clinically important by stakeholders. We have powered this trial to detect a 10% improvement in the primary outcome. To detect a mean difference of 2 days out of ED or hospital at 30 days with a two-sided significance level of 0.05 and a power of 80% with equal allocation to two arms would require 120 patients per group assuming a pooled standard deviation of 5 days. The standard deviation was estimated by assuming the range of measurement will span approximately six standard deviations. Because the primary endpoint is assessed at 30 days and based on the coverage of the EHR, we expect minimal drop-out and missing data. Multiple imputation will be used if more than 20% of patients have missing data on the primary outcome. Aim 3 qualitative inquiry will use a purposeful sampling approach to intentionally select participants who can provide information about their experiences with the program. Based on the past experience of this team, the heterogeneity of expected patient situations, and the scope of this inquiry, we anticipate conducting approximately 25 patient interviews. We anticipate up to 35 clinicians and other stakeholders will also complete aim 3 interviews.

### Recruitment {15}

#### Aim 1

Stakeholders will be identified by the study team and will include individuals who are expected to interact with or be impacted by the CACP program, e.g., potential referring clinicians, members of the health care team (e.g., case managers), CPs, and hospital administrators. Individuals will receive an email informing them of the study and requesting their participation in an interview. Those who respond and indicate interest will be scheduled for an individual interview to be conducted in person or by videoconference. Participants will complete oral consent prior to the interview.

#### Aim 2

Patient identification, enrollment, and consent procedures will be consistent with the pragmatic point-of-care study design and objectives to ensure feasibility within the real-world practice. Consistent with the point-of-care trial approach, the study team will disseminate information about the CACP program and trial to ambulatory, ED, and hospital practices. Clinicians as well as case managers and social workers (who oversee discharge planning in the inpatient and ED settings) will be encouraged to refer patients to the CACP program if this would allow patients to leave the hospital sooner or avoid hospitalization in the first place. The patient’s clinical care team will therefore identify patients for consideration of enrollment into the CACP program based on the patients’ clinical situation and needs. Case managers, social workers, and clinicians will be able to refer patients to the CACP program using established clinical workflows for home health and other post-hospital care programs. Such referrals will automatically result in a notification to the CP team; the CP team will verify clinical eligibility and CACP program capacity. If the request can be accommodated by the CACP program and the program has census capacity for that request, the CP team will then notify the study research team, who will approach the patient for enrollment and informed consent. After informed consent is obtained, the study team member will randomize the patient 1:1 to CACP versus usual care and notify both the CP service and the referring service of the outcome of randomization. If the CACP program has no capacity to accept patients, the referring service will be notified immediately upon the referral. The referring service will then make alternative care plans. This process is analogous to home health agency referrals in the hospital system.

Patients who refuse participation in the CACP trial may be eligible to receive CP services outside of the trial if the number of non-trial patients receiving CP care at that time would be at or below the CACP program capacity prior to the launch of the trial and dedicated CP staffing to support trial activities. In the Rochester location, there was capacity for an average of 1 patient per day (because CPs care for other lower acuity non-trial patient populations as well), while in the Wisconsin location, there was no capacity prior to the trial as the CACP program did not exist there prior to the trial. Patients who consented to participate in the trial but were randomized to usual care will not be able to opt out and request enrollment in the CACP program outside of the trial. However, all patients who complete 30 days of follow-up within the trial will be able to receive CP services after the 30-day period irrespective of their initial randomization allocation. Patients who consented to participate in the trial and were randomized to the CACP arm, and who were hospitalized within their 30-day follow-up period, will be able to receive CP services again upon discharge if clinically appropriate.

#### Aim 3

Patient participants who were randomized to the CACP arm will be contacted by telephone to offer participation in the interview after their last CP visit. Three contact attempts will be made. To ensure diversity of perspectives, the study team will review potential interview participants each week and identify recruitment priorities based on diversity in terms of age (< 50 and ≥ 50 years old), gender, race and ethnicity, rurality, referral geography (WI and MN), diagnosis for which they were referred, referral site (pre-hospital setting or ED or hospital), and whether the patient has an assigned Mayo Clinic PCP.

All CPs who provided CACP care during the trial will be invited by email to participate in an interview. We will also contact a purposive sample of clinicians who referred patients to the CACP program, along with administrators and other members of the health care team for interviews. Referring clinicians will be sampled based on their service line, location (clinic or ED or hospital; Minnesota or Wisconsin), and number of patients referred to the trial. Three contact attempts will be made. Additionally, all CPs, clinicians whose patients were cared for in the CACP study arm, members of the health care team who interacted with the CACP program, and select administrators will receive a survey via email at the end of the trial to seek their feedback on the CACP program. Members of the health care team who interacted with the program will be identified based on data from the CP referral tracker, as well as a review of orders in the EHR.

### Assignment of interventions: allocation

#### Sequence generation {16a}

Individuals will be block randomized 1:1 at the patient level to usual care or to CACP per a computer-generated randomization schedule stratified by source of referral (pre-hospital versus ED versus hospital) and location (Minnesota versus Wisconsin) using blocks of random sizes.

#### Concealment mechanism {16b}

The Remote Electronic Data Capture (REDCap) database software will be used for allocation concealment. The system does not release the randomization code until the participant is enrolled in the trial.

#### Implementation {16c}

The randomization algorithm will be generated by an analyst within REDCap. Stratification factors create six stratum levels as the product of categories of each factor (two geographic locations and three referral sites). Randomization is performed separately within each stratum level, utilizing blocks of random size, based on the schema entered by the data analyst. This sequence is blinded to the individuals entering the patients into the data collection tool. When the patient is identified, consents, and is ready to randomize, the information is entered into the REDCap system with randomization schema, a randomization button is pressed, and REDCap returns the next allocation in the sequence for given strata.

### Assignment of interventions: blinding

#### Who will be blinded {17a}

Participant and care team blinding is not possible in this trial. Study team members collecting qualitative and survey data collection cannot be blinded to study allocation. The statistician will provide unblinded data to the DSMB. The adverse event adjudication team will be blinded to study allocation, though members will have access to the patient’s EHR where CP notes may or may not be present for a given patient. We will use a group nomenclature (group 1 and group 2) during data analysis. The data will be unblinded for appropriate interpretation before publication. Analysis of quantitative data will be performed at the close of the study.

#### Procedure for unblinding if needed {17b}

Neither clinicians nor patients will be blinded to allocation, such that no unblinding will occur.

### Data collection and management

#### Plans for assessment and collection of outcomes {18a}

Participants randomized to CACP will have access to CP services ordered and overseen by the treating clinical team per the current standard of care. Thus, the clinical trial evaluates the CACP care delivery model rather than the clinical care that is provided to patients within it. Participants randomized to usual care will not have access to CP services and will continue with their care as planned by the treating clinician(s).

All participants will complete a baseline survey at the time of enrollment/consent and at 30 days. Participants randomized to CACP will also complete a survey after their last CP encounter. Paper copies of the baseline surveys will be completed at the time of enrollment or provided to the participant and returned by mail. In the CACP treatment arm, we will ask that baseline surveys be completed and returned before or during the first CP visit. In the usual care arm, for participants who did not complete their baseline survey at the time of enrollment, up to 3 reminder phone calls will be made by a member of the research team to participants who have not returned their survey by the end of week 1.

Participants randomized to the CACP treatment arm will also be asked to complete a survey after their last CP visit. This survey will be handed to the participant by the CP at their last visit with a pre-stamped envelope so it can be completely private and returned by mail or sealed and given to the CP for delivery. If participants need help completing the survey, the research team study coordinator will contact them by telephone and complete the survey with them. Patients in the intervention arm who are recruited and consent to participate in an interview will complete it by telephone with a trained member of the study team unaffiliated with intervention delivery. Interviews will be audio-recorded with permission and transcribed for analysis.

Patients in both arms will be asked to complete a paper survey at day 30, administered between days 30 and 40 (first attempt) and with all 3 contact attempts completed by day 90. If participants need help completing the survey, it can be done over the phone with the research team study coordinator.

The EHR will be queried at 30 days, 6 months, and 12 months for ED visits, hospitalizations (excluding planned hospital admissions for scheduled surgeries, procedures, and/or treatments), and other outcomes of interest not collected directly from the patient. The study setting includes two hospital systems with the same Epic EHR and the ability to view records regardless of event location; therefore, we anticipate high completeness of EHR data for this trial. However, we will supplement these data as needed with patient-reported information about ED visits and hospitalizations, ascertained in the 30-day survey and confirmed with phone follow-up.

We will also survey clinicians and other members of the health care team who referred participants enrolled in the CACP intervention arm, administrators involved with the CACP program, and community paramedics and interview purposive samples of clinicians, administrators, and community paramedics. These surveys will be completed using a web-based survey platform. Survey invitations will be sent by email, and non-responders will receive up to three reminder contacts. Interviews will be conducted in-person or by telephone or video conference, and audio recorded and transcribed for analysis.

#### Plans to promote participant retention and complete follow-up {18b}

For all patient surveys and interview invitations, up to three contact attempts will be made to ensure the completion of surveys and/or interviews. For stakeholder and CP interviews and surveys, potential participants will be contacted up to three times to inquire about interview and survey participation.

#### Data management {19}

Data will be collected directly from the patient or from the Epic EHR system. REDCap will be used to log electronic data collected from the Epic EHR. REDCap is a HIPAA-compliant database that contains multiple levels of authentication and access controls. All changes to the data and each “record viewed” are logged back to the individual login ID with a timestamp. REDCap also has several built-in functions to control the exportation of identifying information.

Medical records will be abstracted electronically by approved research staff. Paper forms (if necessary) may also be uploaded as a PDF or manually entered into the REDCap system. All research material will be maintained on a secure server or locked in file cabinets. All material will be destroyed 7 years following the completion of the study. The research data are only accessible with password-protected and logged access to a secure server at Mayo Clinic. Other study data, e.g., interview data, will be stored in a secured server with restricted access only to the study staff. While qualitative data will not be made available on an open platform, published results will include a rich description that includes de-identified quotes from participants.

#### Confidentiality {27}

The privacy of all study participants will be protected by avoiding the use of names on all research data. All study participants will be identified by a unique study code number. The link between the code number and the study participant’s identity will be stored in a remote data capture (RDC) system that is password-protected and only accessible to study personnel, all of whom have human subject research training.

The potential risks of breached confidentiality will be minimized by ensuring that the data are private, only used by members of the research team, and only used for the specific purposes of this study.

#### Plans for collection, laboratory evaluation, and storage of biological specimens for genetic or molecular analysis in this trial/future use {33}

This trial does not involve the collection, laboratory evaluation, or storage of biological specimens for genetic or molecular analysis.

## Statistical methods

### Statistical methods for primary and secondary outcomes {20a}

#### Aim 1

We will use methods of rapid qualitative analysis, guided by constructs in the Consolidated Framework for Implementation Research (CFIR) [22]. Members of the study team will meet to review notes and audio recordings from interviews and discuss issues that may impact implementation, including those that should be addressed before trial enrollment commences. Notes will be summarized in a template guided by the CFIR framework and imported into qualitative software (NVivo, QSR International) for data management. Summaries will be presented at biweekly study team meetings for discussion of potential actions, including changes to the implementation plan.

#### Aim 2

The main analysis of the primary outcome will be based on the intention-to-treat (ITT) principle, with all patients randomized to CACP compared to all patients randomized to usual care. The ITT sample will comprise all patients who were randomized and completed the baseline assessment. The primary outcome (CACP effectiveness) is defined as the number of days the patient was alive and outside of the ED or hospital (excluding planned hospital admissions for scheduled surgeries, procedures, and/or treatments). Outcomes will also be analyzed in the per-protocol subset. The per-protocol sample will comprise all patients in the ITT arm except for those who did not follow protocol, i.e., patients who did not receive CP services in the intervention arm.

The mean number of days out of the ED or hospital for the intervention group will be compared to that of the control arm. Statistical significance, and a two-sided confidence interval for the difference between arms, will be assessed by using the two-sample *t* test. The distributional assumptions for the test will be assessed by calculating the skew and kurtosis for the bootstrap distribution of the sample difference between means. The Hodges-Lehman shift estimator for the Wilcoxon rank-sum test will be used if the absolute value of the skew or kurtosis-minus-three are greater than 1.5.

Other continuous outcome measures will be assessed by the same method as the primary outcome measure. Categorical measures will be assessed by using the Pearson chi-square test, or the Fisher exact test will be used if the minimum expected cell count is less than five.

Sensitivity analyses concerning the primary and secondary endpoints will be conducted, including excluding hospice deaths, examining the impact of the number of CP visits, examining the impact of the duration of CP care (< 10 days, 10–19 days, 20–29 days) and per protocol vs ITT. The impact of covariates will be assessed by using multiple linear regression or multiple logistic regression.

Descriptive statistics and figures will be provided as appropriate for secondary outcomes, and baseline demographics will be summarized for each arm. Validated instruments (e.g., EQ-5D) will be scored per official guidance, and the results will be reported as appropriate. When data is available, changes in rates or scores between baseline and 30 days will be reported for each arm along with comparisons between the groups. Implementation outcomes will be summarized using descriptive statistics.

#### Aim 3

Qualitative data will be analyzed following qualitative content analysis procedures, guided by the theoretical constructs employed in the study. Analysis will begin with at least two members of the study team reviewing audio files and transcripts. A coding framework will be developed, starting with a priori topics in the interview guide and constructs from theoretical frameworks like CFIR. Analytic memos will be used to summarize the findings, including any key differences by participant role.

Surveys will be analyzed using descriptive statistics and scoring guidance for validated instruments (e.g., CSAT). Integration of findings will involve a comparison of quantitative and qualitative findings by construct, e.g., survey and interview data on acceptability and sustainability.

### Interim analyses {21b}

There will be no interim analysis of effectiveness or implementation outcomes for the purpose of early stopping of the trial. Interim analyses will be limited to those completed for safety monitoring. They will be conducted by the study statistician, and the results will be made available to the Data and Safety Monitoring Board (DSMB). The results will not be blinded to the DSMB as they will be used to ensure safety of continuing the trial. Analyses will be conducted independently of the investigators.

### Methods for additional analyses (e.g., subgroup analyses) {20b}

Heterogeneity of treatment effects analyses will be completed for the subgroup categories of patient age (< 45 years, 45–64 years, ≥ 65 years), patient gender (male, female), patient race/ethnicity (non-Hispanic White, non-White), patient rurality (rural, non-rural), referral source (pre-hospital, ED, hospital), reasons for referral, and types of services delivered (as indicated in the patient referral tracker). The analyses will be performed for the primary outcome measure by using a general linear model with terms for arm, subgroup category, and treatment by subgroup category interaction.

### Methods in analysis to handle protocol non-adherence and any statistical methods to handle missing data {20c}

Primary analyses will be conducted using an intention-to-treat approach. Secondary analysis will be conducted using the per-protocol approach, where patients randomized to the CACP arm who never received the intervention or did not complete the intervention (i.e., did not respond to CP service calls to be scheduled and had to be administratively discharged from the CACP program) will be excluded. Patients randomized to the control condition will not be eligible to receive CP services during the 30-day follow-up period. We will confirm this using an EHR review and exclude any patients who received CP care during that period.

Missing data: For the primary outcome, missing data (e.g., loss to follow-up, withdrawal, death) are expected to be minimal at 30 days, as data are collected from the EHR and are part of the standard of care. Primary analyses will be conducted on the intention-to-treat population. For missingness greater than 20%, multiple imputation will be used.

If patients self-report on *T*_3_ surveys any hospitalizations or ED visits not captured in the EHR, the study staff will confirm those visits using phone follow-up. We will report the percentage of missing data for all primary and secondary outcomes at each time point, as well as for individual survey items. For validated measures with multiple items, we will follow scoring guidance for missing data.

### Plans to give access to the full protocol, participant-level data, and statistical code {31c}

The full protocol, de-identified datasets, and statistical code may be made available from the corresponding author upon reasonable request and appropriate resources after the final publication of results.

### Oversight and monitoring

#### Composition of the coordinating center and trial steering committee {5d}

The trial will have oversight by a steering committee, which includes the principal investigator and co-investigators. The steering committee is responsible for reviewing and approving any modifications to the study protocol. The committee will meet monthly to review the study progress, including trial recruitment. They will also review data on three categories of adverse events (AE) and report serious adverse events (SAE) to the DSMB and IRB.

#### Composition of the data monitoring committee, its role, and reporting structure {21a}

This study will use a DSMB that is independent from the study sponsor and investigator team. Its members represent diverse areas of expertise that are key components of the proposed study, specifically emergency, hospital medicine, and biostatistics. The DSMB will meet via videoconference every three months.

#### Adverse event reporting and harms {22}

The study team will monitor for AE in both groups including ED visits, unplanned hospitalizations, deaths, falls with injury, and medication errors. SAEs for this study are any events that result in death, are life-threatening or place the participant at immediate risk of death from the event as it occurred, result in an ED visit or hospitalization, or cause persistent or significant disability or incapacity. The study statistician will check for hospitalizations, ED visits, and deaths on a weekly basis. Additionally, study coordinators review patient charts over the course of the study volunteer period, ensuring all adverse events are properly reported to the IRB as required per protocol. AEs and SAEs will be tracked in an Excel adverse event tracking log by the study research coordinators. AE and SAE that occur will be reviewed by the adjudication committee, which is comprised of clinical experts in hospital medicine, emergency medicine, cardiology, primary care, and paramedicine. The principal investigator is not a member of the adjudication committee.

The adjudication committee will assess the relationship of all AEs to the study intervention, based on temporal relationship and clinical judgment. The degree of certainty about causality will be graded using the categories below.

- Definitely related—There is clear evidence to suggest a causal relationship, and other possible contributing factors can be ruled out. The clinical event, including an abnormal laboratory test result, occurs in a plausible time relationship to study intervention administration and cannot be explained by concurrent disease or other drugs or chemicals. The response to the withdrawal of the study intervention (dechallenge) should be clinically plausible. The event must be pharmacologically or phenomenologically definitive, with the use of a satisfactory rechallenge procedure if necessary.
- Possibly related—There is some evidence to suggest a causal relationship. However, other factors may have contributed to the event (e.g., the participant’s clinical condition, other concomitant events). Although an AE may rate only as “possibly related” soon after discovery, it can be flagged as requiring more information and later be upgraded to “probably related” or “definitely related,” as appropriate.
- Not related—The AE is completely independent of study intervention, and/or evidence exists that the event is definitely related to another etiology. There must be an alternative, definitive etiology documented.

All AEs related to the intervention will be reviewed in duplicate by two members of the adjudication committee, and their severity will be graded using the following guidelines:

- Mild–events that require minimal or no treatment and do not interfere with the participant’s daily activities.
- Moderate—events that result in a low level of inconvenience or concern with therapeutic measures. Moderate events may cause some interference with functioning.
- Severe—events that interrupt a participant’s usual daily activity and may require systemic drug therapy or other treatment. Severe events are usually potentially life-threatening or incapacitating. Of note, the term “severe” does not necessarily equate with “serious.”

Disagreements in adjudication will be reviewed by a different member of the adjudication committee. Final adjudication results will be compiled by the study coordinator and used for analysis and DSMB reporting purposes.

Throughout the course of the study, we will also monitor AEs in both study arms and may suspend accrual if patients are experiencing a large number of adverse events. In particular, we will temporarily suspend accrual if, at any time, observed events meet the following criteria: (1) If, within the first 30 patients, 2 or more patients in the CACP arm than the usual arm experience a SAE, the study will be paused for review by the DSMB; (2) if, after the first 30 patients, the proportion of participants in the CACP arm who experience an SAE is ≥ 10% greater than the proportion of participants experiencing SAE in the usual care arm, the study will be paused for review by the DSMB.

If unexpected adverse event profiles are observed which do not meet the above criteria, but are worrisome, we may still consider temporarily suspending accrual. Adverse event-stopping rules may be modified if, during the study, new information becomes available, which suggests that such a modification is necessary, or, if after temporarily stopping accrual, the above rule is found to be overly conservative.

Reports containing efficacy, AE, and administrative information will be compiled and presented to the DSMB every three months.

#### Frequency and plans for auditing trial conduct {23}

The principal investigator, project manager, and study coordinators will be responsible for monitoring study quality, including the accuracy of data entered in REDCap. The IRB will continue to review the trial and approve consent documents and protocols at least annually. The study coordinator checks consent forms, compliance with the protocol and the planned interventions, and the quality of survey data. The study sponsor receives quarterly progress reports but does not participate in audit activities.

#### Plans for communicating important protocol amendments to relevant parties (e.g. trial participants, ethical committees) {25}

Important protocol modifications (e.g., changes to eligibility criteria, outcomes, analyses) and protocol deviations will be communicated to the steering committee. Modifications to human subject research procedures and protocol deviations will be reviewed by the IRB as appropriate. The IRB will make determinations as to whether participants should be contacted regarding amendments.

#### Dissemination plans {31a}

Research findings will be presented at conferences, published in peer-reviewed journals, and shared with the practice. If the results are favorable to the intervention, we will work with relevant stakeholders to expand access to the intervention within the organization and to share details of the intervention with other health care organizations.

## Discussion

This novel hybrid type 1 pragmatic trial will assess the effectiveness and explore the implementation of the CACP program within diverse real-world settings. Pre-implementation work is aimed at minimizing practice interruption and ensuring feasible trial conduct. Mixed methods with a range of stakeholders, including patients, will better ensure that multiple perspectives are represented and that the study team can rapidly move to scale up if the intervention is effective. The study team anticipates being able to enroll patients and conduct primary analyses within 1 year to meet the care and information needs of the clinical practice.

## Trial status

This trial is currently ongoing. Recruitment and allocation of patients began on January 21, 2022. The current trial protocol is version 4 (approved June 10, 2022). The planned end of recruitment is December 31, 2022.

## Data Availability

The full protocol, de-identified datasets, and statistical code may be made available from the corresponding author upon reasonable request and appropriate resources after the final publication of results. There are no contractual agreements that limit such access for investigators.

## Abbreviations

AE: Adverse event
CACP: Care Anywhere with Community Paramedics
CFIR: Consolidated Framework for Implementation Research
CP: Community paramedic
CSAT: Clinical Sustainability Assessment Tool
DSMB: Data Safety Monitoring Board
EMS: Emergency medical services
ED: Emergency department
EHR: Electronic health record
IRB: Institutional Review Board
IV: Intravenous
LOS: Length of stay
PCP: Primary care provider
SAE: Serious adverse event
SNF: Skilled nursing facility
